# Real-time optical imaging of the hypoxic status in hemangioma endothelial cells during propranolol therapy

**DOI:** 10.3389/fonc.2022.995745

**Published:** 2022-10-04

**Authors:** Yue Wu, Xiaojuan Yang, Mingrui Zhai, Yi Chen, Xiaoya Lu, Jiandong Ju, Huanqing Zhang, Guanduo Wang, Zhe Zhang, Baocun Zhu, Xuan Wang, Zhanwei Chen, Shengyun Huang

**Affiliations:** ^1^ Department of Oral and Maxillofacial Surgery, Shandong Provincial Hospital, Shandong University, Jinan, China; ^2^ Department of Orthodontics, School and Hospital of Stomatology, Cheeloo College of Medicine, Shandong University, Jinan, China; ^3^ School of Water Conservancy and Environment, University of Jinan, Jinan, China; ^4^ Department of Oral and Maxillofacial Surgery, Shandong Provincial Hospital Affiliated to Shandong First Medical University, Jinan, China

**Keywords:** hemangioma, propranolol therapy, fluorescent probe, fluorescence imaging, hypoxic status

## Abstract

Infantile hemangioma (IH) is the most common microvascular tumor of infancy involving the area of head and neck. One of the most important independent risk factors of IH is the hypoxia microenvironment. Fluorescent chemosensor provides a noninvasive intervention, high spatiotemporal resolution, ultrasensitive response, and real-time feedback approach to reveal the hypoxic status of cells. Our research group developed an ultrasensitive fluorescent chemosensor, HNT-NTR, and investigated the potential ability of imaging the hypoxic status of hemangioma-derived endothelial cells (HemECs). In this study, we successfully visualized the propranolol (PRN) treatment in HemECs using NHT-NTR with “Turn-off” sensing method. This chemosensor exhibited high sensitivity and selectivity for optical imaging of hypoxic status with fast responsiveness, real-time feedback and durable photostability of the fluorescent signal. It was also confirmed that HNT-NTR could monitor nitroreductase *in vivo*. Paramountly, we expected this chemosensor to offer an available optical method for imaging of the hypoxic status and visualizing the therapeutic status of PRN therapy in IH with the hypoxia-imaging capability.

## Introduction

Infantile hemangioma (IH) is the most common microvascular tumor in infancy and mostly involves head and neck area (1). IH presents as a red, lobulated plaque in the superficial dermis or as a light blue mass subcutaneously (2). It has been reported that the incidence of IH ranges from 2-10% (3). Hypoxia and the renin-angiotensin system have been demonstrated as independent risk factors in the etiology of IH. Tan’s group has postulated that the hypoxia condition of infants with prematurity and low birthweight is a contributor to the higher incidence of IH, shedding light on the potential linkage between hypoxia and the occurrence of IH (4). The increment of hypoxia inducible factor-1α (HIF-1α) positive nuclei in both endothelial and interstitial cells in proliferating IH has also been demonstrated (5). However, the enigmatic role that hypoxia plays in the pathogenesis and clinical treatment of IH still needs further investigation.

For detecting hypoxic status, special facilities or methods have been developed. For example, oxygen microelectrodes have been designed for the measurement of dissolved oxygen (6), which is also considered a golden standard in detecting hypoxic status. Whereas, this technology requires an invasive penetration of the microelectrode. Other facilities like BOLD (Blood Oxygen Level Dependent) functional MRI (7), PET (8), and SPECT (9) are noninvasive approaches for hypoxic tissue identification. However, the radiolabeled molecular agents and the poor tumor-to-background (TBR) level have hindered their clinical usage (10). As for detecting the hypoxic status in IH, the most regular methods are real-time RT-PCR, western blotting, and immunohistochemistry to evaluate hypoxia-related biomarkers (11–13), such as HIF-1α, instead of the hypoxic status itself. When compared to the conventional detecting facilities or methods above, fluorescent probes, also known as fluorescent chemosensors (14) are becoming increasingly important, providing noninvasive intervention, high spatiotemporal resolution, ultrasensitive response, and real-time feedback (15, 16). A hypoxia- activable fluorescent chemosensor might become an available option for detecting the hypoxic status in IH.

Some clinical therapies for IH have also been demonstrated to change the progression of the disease depending on some hypoxia-related mechanisms. It has been reported that one of the first-line treatment drugs for IH, propranolol (PRN), can suppress IH proliferation, migration and tube formation through HIF-1α dependent pathway (17). PRN could also inhibit the activity of HIF-1α (18) and suppress the HIF-1α-VEGF-A angiogenesis axis in IH (19). Since HIF-1α is one of the essential transcription factors in hypoxia (20), the results implied the potential effect of PRN to change the hypoxic microenvironment in IH indirectly. However, whether PRN could modify the hypoxic status in hemangioma endothelial cells (HemECs) requires further investigation.

In the past few years, hypoxia-activable fluorescent chemosensors have moved into the spotlight for monitoring hypoxic conditions. Some overexpressed enzymes in hypoxic status, such as nitroreductase (NTR), have become an appropriate response method for hypoxia-activable fluorescent chemosensors (21) (22, 23). Nonetheless, the majority of the newly developed fluorescent chemosensors are concentrated on malignant tumors. To our knowledge, none of them were applied in hemangioma. On the other hand, most of the experiments elucidating the potential pathogenic linkage between hypoxia and hemangioma at present use conventional methods mentioned above (11–13). The potential utilization of hypoxia-activable fluorescent probes with NTR-responsive methods merits further investigation for fluorescence imaging of both the dynamic modification during treatment and the undynamic hypoxic status in HemECs.

**Scheme 1:**
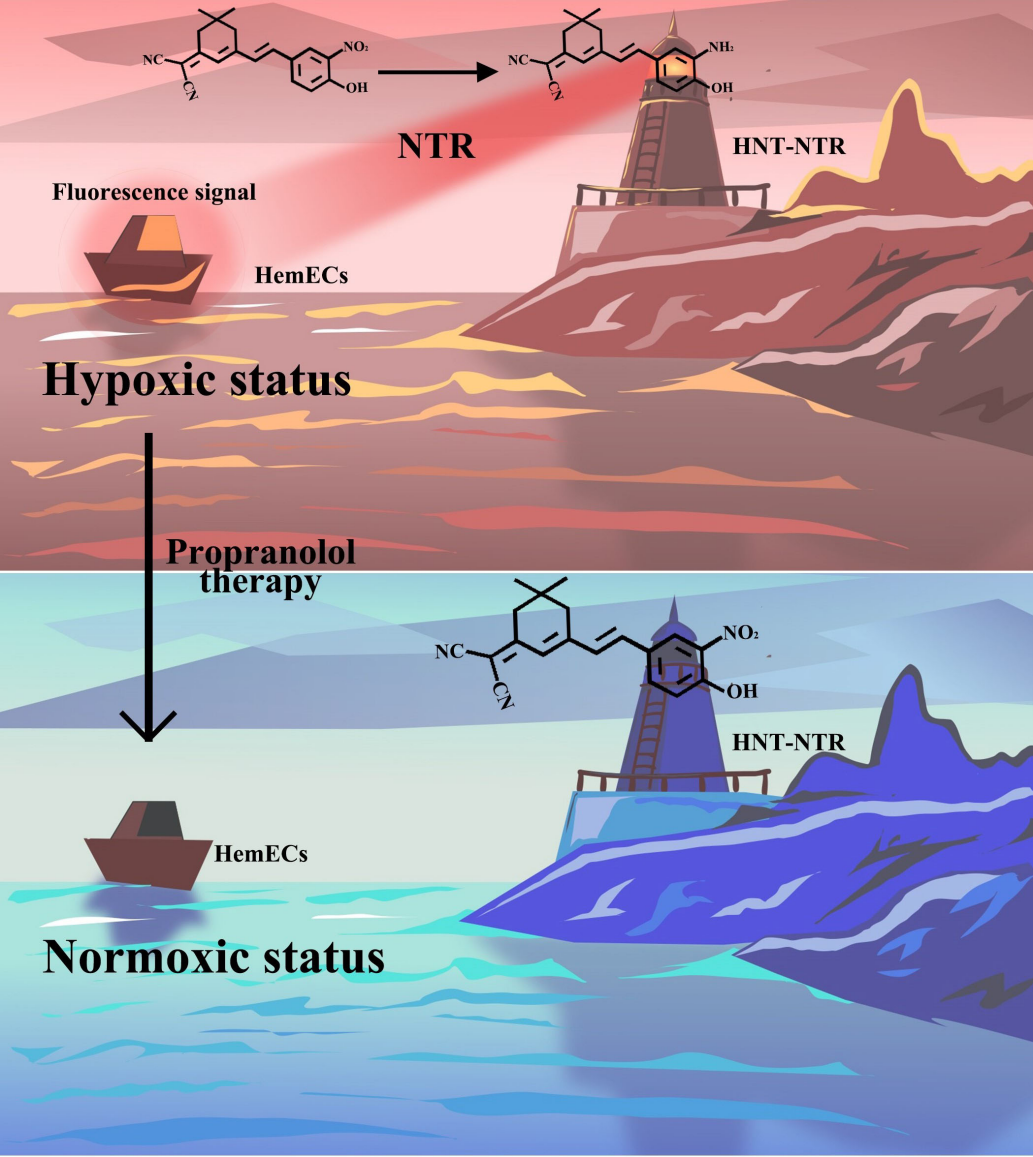
Schematic illustration of the response mechanism of HNT-NTR and the optical imaging of the hypoxic status during propranolol therapy. The novel fluorescence chemosensor HNT-NTR could visualize the change of hypoxic status during PRN treatment in HemECs by “Turn-off” sensing method with high biocompatibility and durable photostability.

Recently, our research group has synthesized an ultrasensitive near-infrared fluorescent probe, HNT-NTR, and used it to distinguish tumor cells from normal cells and compared the invasiveness of different tumor cell types (24). In this article, we made a further step to visualize the change of hypoxic status during PRN treatment in HemECs with NHT-NTR by "Turn-off" sensing method, and the process of it was shown in **Scheme 1**. After being treated with PRN in low concentrations, HemECs in hypoxic status showed a significantly inhibited fluorescence signal in real-time. We also identified that NHT-NTR could visualize the hypoxic status of HemECs effectively with high biocompatibility and durable photostability. Our results pave a new way for optical imaging of the hypoxic status in HemECs with a novel fluorescence chemosensor. Conclusions could be made that PRN could modify the hypoxic status of HemECs and the novel chemosensor NHT-NTR could visualize such a modification with high sensitivity and selectivity during PRN therapy.

## Materials and methods

### The synthesis and chemical characterization of HNT-NTR

The detailed synthesis process, chemical response mechanism and chemical characterization could be found in our previous achievement (24). We also demonstrated the synthesis process in the **Supplementary Material** for convenience.

### Cell culture

HemECs were cultured in endothelial cell medium, containing 10% fetal bovine serum (FBS), 1% penicillin-streptomycin, and 1% Epidermis Growth Factor (EGF) at 37 °C under 5% CO_2_. The medium was changed every other day. The detailed process of isolation and culture of HemECs could be found in the **Supplementary Material**.

### Fluorescence imaging of the hypoxic status in living HemECs

HemECs were firstly cultured in Endothelial Cell Medium (ECM) with 5% FBS, 1% Penicillin-Streptomycin and 1% EGF at 37 °C under 5% CO_2_ atmosphere for 2 days. To investigate the potential ability to visualize hypoxic status in hemangioma, HemECs were seeded in 24-well plates (8000/mL, 1 mL per well) and then cultured at 37 °C, under 5% CO_2_ atmosphere for 1 day. After that, the plates were divided into 2 groups: normoxia group and hypoxia group. The plates in normoxia group were then cultured in regular atmosphere (21% O_2_), and the ones in hypoxia group were cultured in hypoxic atmosphere (0.1% O_2_) for 48 hours. After washing 3 times with PBS, both the cells in 2 groups were incubated with 30 μM HNT-NTR for 75 minutes. After washing 3 times with PBS, the cells were covered by 200 μL ECM to maintain the biological activity of living cells. Finally, the fluorescence signals were observed in ZEISS inverted microscope (Zeiss Axio Vert, Germany).

### Dicoumarin inhibitory test in living HemECs

Since the underlying mechanism of this hypoxia-activable fluorescent chemosensor is based on the overexpressed NTR, we further identified the response effect of HNT-NTR reversely with a typical NTR inhibitor dicoumarin (10, 25). HemECs were seeded on the cell slides in 24-well plates (8000/mL, 0.5 mL per well) and then cultured at 37 °C, under 5% CO_2_ atmosphere for 1 day. Then, HemECs were cultured in hypoxic condition (0.1% O_2_) and normoxic condition respectively for 48 hours. Next, the HemECs in hypoxic condition were divided into 2 groups: dicoumarin blockage group and hypoxia group. The cells in dicoumarin group were pretreated with 1 mM dicoumarin for 1 hour. After washing 3 times with PBS, all the groups were then co-incubated with HNT-NTR (30 μM, 0.5 mL) for 75 minutes. These HemECs were fixed with 90% ethanol for 20 minutes and then stained with 200 μL DAPI for 10 minutes. Finally, we picked up the inverted cell slices and placed them in glass slides with antifading fluorescent mounting medium and the fluorescent signals were observed by confocal fluorescence microscopy (Leica TCS SP8 MP).

### Real-time detection and photostability in living HemECs

To evaluate the response speed and the real-time feedback capability of HNT-NTR, HemECs were cultured in the same procedure as 2.4. After that, both hypoxia group and normoxia group were firstly observed in ZEISS inverted microscope (Zeiss Axio Vert, Germany) before the treatment of HNT-NTR. After adding HNT-NTR, the cells in the same locations were then observed again immediately for comparison to show the real-time changes of fluorescence signals. To estimate the time-dependent fluorescence signals of HNT-NTR, HemECs were pre-cultured in the hypoxic atmosphere (0.1% O_2_) for 48 hours. After washing 3 times with PBS, HNT-NTR was added for 75 minutes, and 4% paraformaldehyde was used for 15 minutes to stable the cell morphology. After washing with PBS 3 times, 500 μL PBS was added to each plates for observation. Fluorescence signals were finally collected at the time point of 20, 40, 60, 80, 100, 120, 140, 160, and 180 minutes.

### 3D microsphere imaging of hemangioma

To prepare 3D microspheres of hemangioma, HemECs were seeded in 96-well round bottom ultralow attachment spheroid microplate at a density of 1×10^5^ cells/mL, 100 μL/well, and then cultured in 37 °C, under 5% CO_2_ for 2 days. After the formation of microspheres, the cells were cultured in the hypoxia atmosphere (0.1% O_2_) for 48 hours. The microspheres were then divided into 3 groups: Dicoumarin group, probe group and control group. As for dicoumarin group, microspheres were firstly pretreated with 1 mM dicoumarin and then co-incubated with 40 μM HNT-NTR for 75 minutes. As for probe group, microspheres were treated with 40 μM HNT-NTR for 75 minutes. As for control group, microspheres were only cultured in ECM. After washing 3 times with PBS, the microspheres were covered by 100 μL ECM to maintain the biological activity and the fluorescence signals were observed in ZEISS inverted microscope (Zeiss Axio Vert, Germany).

### Biocompatibility and biotoxicity evaluation

To evaluate the biocompatibility of HNT-NTR, HemECs were measured with Cell Counting Kit-8 (CCK-8) assay. We achieved the OD values and transformed them into cell viabilities. The detailed process could be found in the **Supplementary Files**

### *In vivo* imaging of HNT-NTR

The embryos were firstly cultured in E3 embryo media for 5 days. The 5-days-old zebrafish were then kept in 96 well-plates and divided into 3 groups: Dicoumarin group, probe group and control group. Zebrafish in dicoumarin group were firstly pretreated with 100 μM dicoumarin and then co-incubated with 30 μM HNT-NTR (dissolved in E3 embryo media) for 40 minutes. Zebrafish in probe group were treated with 30 μM HNT-NTR (dissolved in E3 embryo media) for 40 minutes. Zebrafish in control group were only cultured in E3 embryo media.

### Imaging of the hypoxic status in HemECs during PRN therapy

To identify the potential modification of the hypoxic status during PRN therapy and expand the potential application of HNT-NTR, HemECs were cultured on the cell slides in 24-well plates (5000/mL, 1 mL per well) and then cultured at 37 °C, under 5% CO_2_ atmosphere for 2 days. Then, HemECs were incubated with 50 μM, 10 μM PRN, and pure ECM (0 μM) respectively and cultured at 37 °C, under 5% CO_2_ atmosphere for 1 hour. After that, the cells were washed with PBS 3 times and co-cultured with 40 μM HNT-NTR for 45 minutes. These HemECs were next fixed with 4% paraformaldehyde for 20 minutes to maintain the real-time biological status after treatment for better observation. The cells were then stained with 300 μL DAPI for 15 minutes to localize the cells when observed by confocal microscopy. Finally, we picked up the inverted cell slices and placed them in glass slides with antifading fluorescent mounting medium and the fluorescent signals of each concentration were observed by confocal fluorescence microscopy (Leica TCS SP8 MP).

## Results and discussions

### Synthesis of HNT-NTR and characteristics evaluation

The process of synthesis has been demonstrated in our previous studies. The spectrophotometric experiment identified the spectra of HNT-NTR towards NTR. In the selectivity test, a remarkable increment of fluorescence intensity was observed after adding NTR, whereas other biological relevant species induced little change in fluorescence intensity. The results indicated that HNT-NTR had high selectivity to NTR compared to other potential species. A detailed illustration could be found in our previous article (24).

### Imaging of the hypoxic status in HemECs

**Figure 1:**
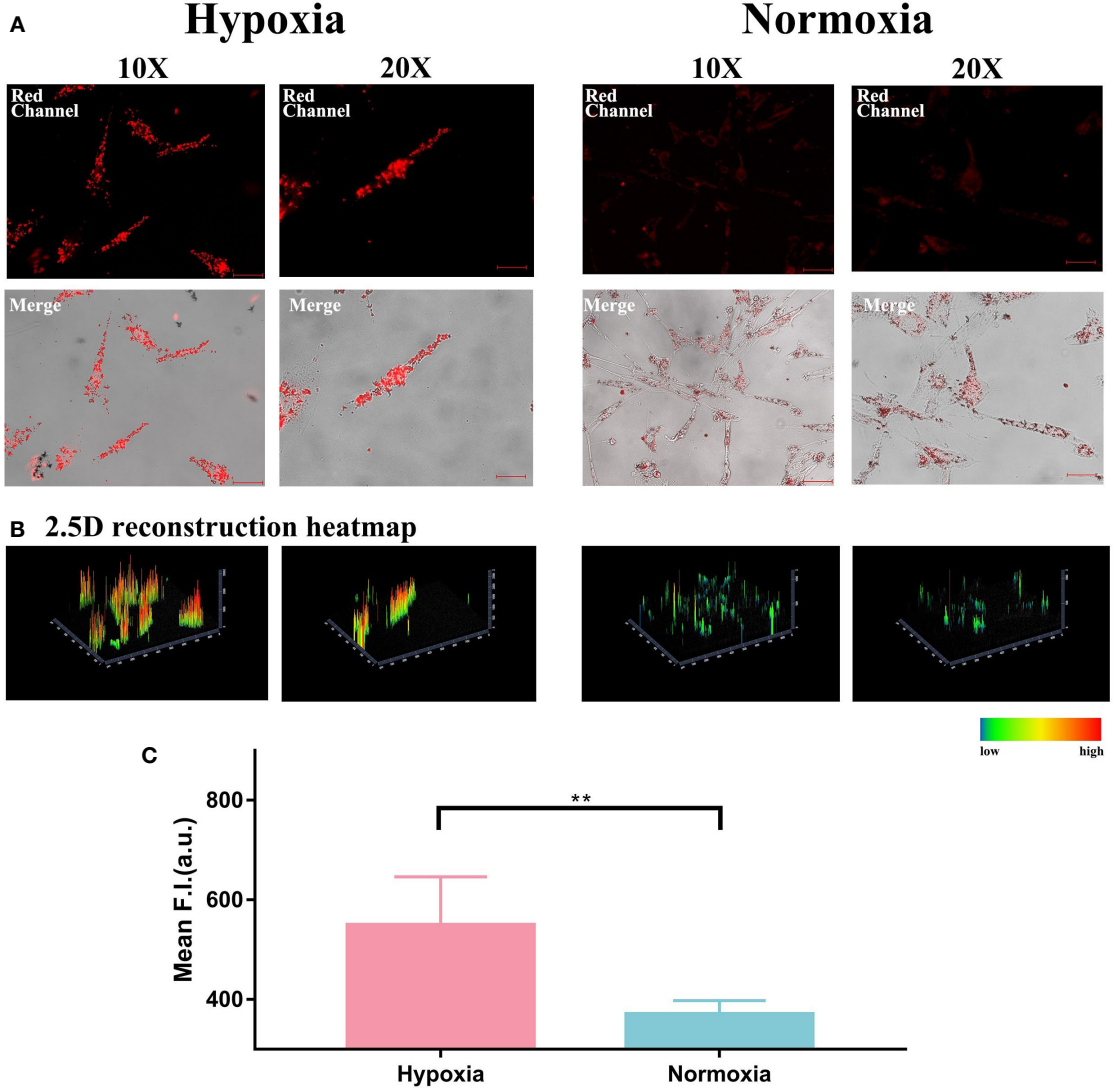
**(A)** Visualization of hypoxic status and normoxic status in living HemECs using HNT-NTR. HemECs were cultured in normoxic (21% O2) and hypoxic (0.1% O2) atmospheres for 48 hours and then co-incubated with HNT-NTR. The cells in hypoxic status showed higher fluorescence signal than in normoxic status. **(B)** 2.5D reconstraction heatmap of the fluorescence signal in each image. We used 2.5D reconstraction heatmaps to transfer the fluorescence intensity of each position into the height of the histogram and the difference of the pseudo-color in the scale bar straightforwardly. The 2.5D heatmap of hypoxia group exhibited higher bars and more red area compared to normoxia group. **(C)** Quantitative analysis of the regions of interest (ROI). We used Zen Blue Lite for the quantitative analysis of fluorescence intensity in regions of interest (ROI) and analyzed the data in GraphPad Prism 7. The fluorescence intensity in hypoxia group was 549.2±43.34 compared to 370.4±12.24 in normoxia group, P<0.01. Scale bar= 50 μm (10X); 25 μm (20X). **:P≤0.01.

Firstly, we cultured HemECs in both normoxic (21% O_2_) and hypoxic (0.1% O_2_) atmosphere for 48 hours and then treated them with HNT-NTR as mentioned in 2.3. As shown in **Figure 1A**, the cells in hypoxic status showed higher fluorescence signals than in normoxic status. Furthermore, the 2.5D heatmap was also reconstructed in Zen Blue Lite, which can transfer the fluorescence intensity of each position into the height of the histogram and the difference of the pseudo-color in the scale bar straightforwardly. As displayed in **Figure 1B**, the 2.5D heatmap of hypoxia group exhibited higher bars and more red area compared to normoxia group. After that, the quantitative analysis of fluorescence intensity in regions of interest (ROI) was calculated in Zen Blue Lite and analyzed in GraphPad Prism 7, which was shown in **Figure 1C**, indicating a significant difference between the two groups (549.2 ± 43.34 in hypoxia group versus 370.4 ± 12.24 in normoxia group, *P*=0.004). The results demonstrated that HNT-NTR could visualize the hypoxic status in living HemECs.

**Figure 2:**
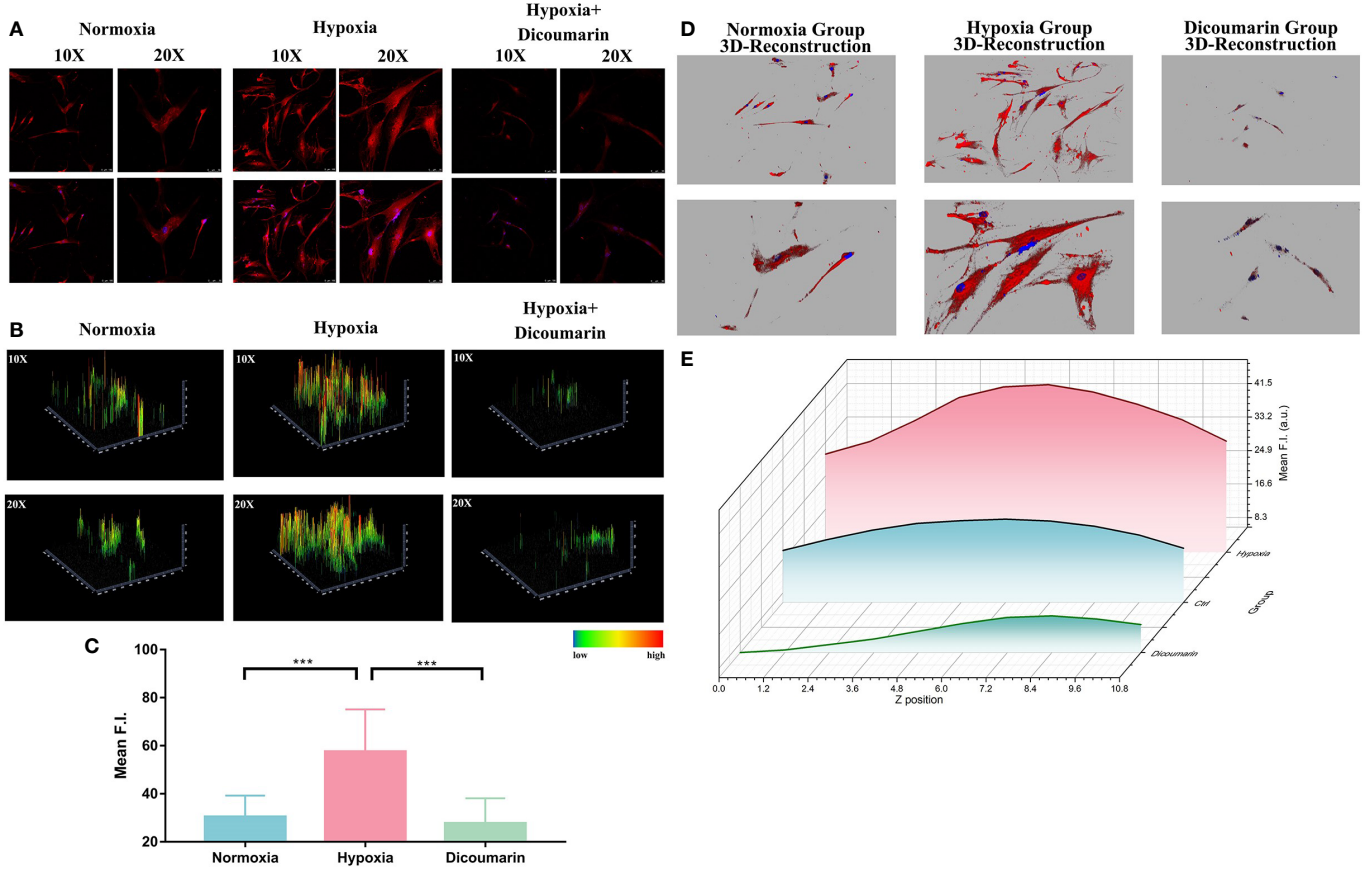
**(A)** Fluorescence imaging of the hypoxia blockage test. HemECs were pre-cultured in hypoxia atmosphere for 48 hours. After adding dicoumarin, the fluorescence signal was significantly inhibited. The upper images refer to the fluorescence signal of HNT-NTR, and the buttom ones refer to the merged signal of both HNT-NTR and DAPI for localization of HemECs. **(B)** 2.5D reconstraction heatmap of the fluorescence signal in each image. The 2.5D heatmap of hypoxia+ dicoumarin group exhibited lower bars and more green/dark area compared to hypoxia group. **(C)** Quantitative analysis of the regions of interest (ROI). The fluorescence intensity of dicoumarin group (27.71 ± 3.463) was significantly lower than the hypoxia group (57.52 ± 5.866, P<0.001) and a bit lower than the normoxia group (30.35 ± 2.966) with no statistical differences. **(D)** 3D reconstruction model of each group. We scanned z axis and transformed the 2D monolayer into a 3D model to reveal the overall fluorescence imaging effect in every z axis position. **(E)** Z axis scanning and quantitative analysis of fluorescence intensity in each cross section. HNT-NTR permeated the whole cytoplasm for visualizing the hypoxic status and the 3D scanning could reduce errors caused by the excessive cell volume effectively. Scale bar= 50 μm (10X); 25 μm (20X). ***P≤0.001.

Because the response strategy of this hypoxia-activable fluorescent chemosensor is based on the overexpressed NTR in cells in hypoxic status. we also used NTR inhibitor dicoumarin for blockage test. As shown in **Figure 2A**, dicoumarin reduced the fluorescence signal. Although the HemECs were pre-cultured in hypoxic condition for 48 hours, after adding dicoumarin, the fluorescence signal was significantly inhibited. The 2.5D reconstruction heatmaps in **Figure 2B** were also in accordance with the result above. After that, the quantitative analysis of ROI was carried out and indicated the significant difference between the hypoxia group (57.52 ± 5.866) and both the normoxia group (30.35 ± 2.966) and blockage group (27.71 ± 3.463) according to **Figure 2C**, hinting the responsiveness of HNT-NTR to NTR was quite sensitive in reverse.

Besides, due to the large size of HemECs, the z-axis localization of confocal microscopy would influence the received fluorescence signal. To calculate the fluorescence intensity meticulously in a 3D manner and restore the overall fluorescence imaging effect in every z-axis position, we scanned z-axis and transformed the 2D monolayer into a 3D model, which was shown in **Figure 2D**. The fluorescence intensity in each cross section was calculated (**Figure 2E**) and the fluorescence signal in probe group was the highest. The results indicated that HNT-NTR permeated the whole cytoplasm for imaging the hypoxic status in HemECs and the 3D scanning test could reduce errors caused by the excessive cell volume effectively.

### Investigation of the real-time detecting ability and photostability of HNT-NTR

**Figure 3:**
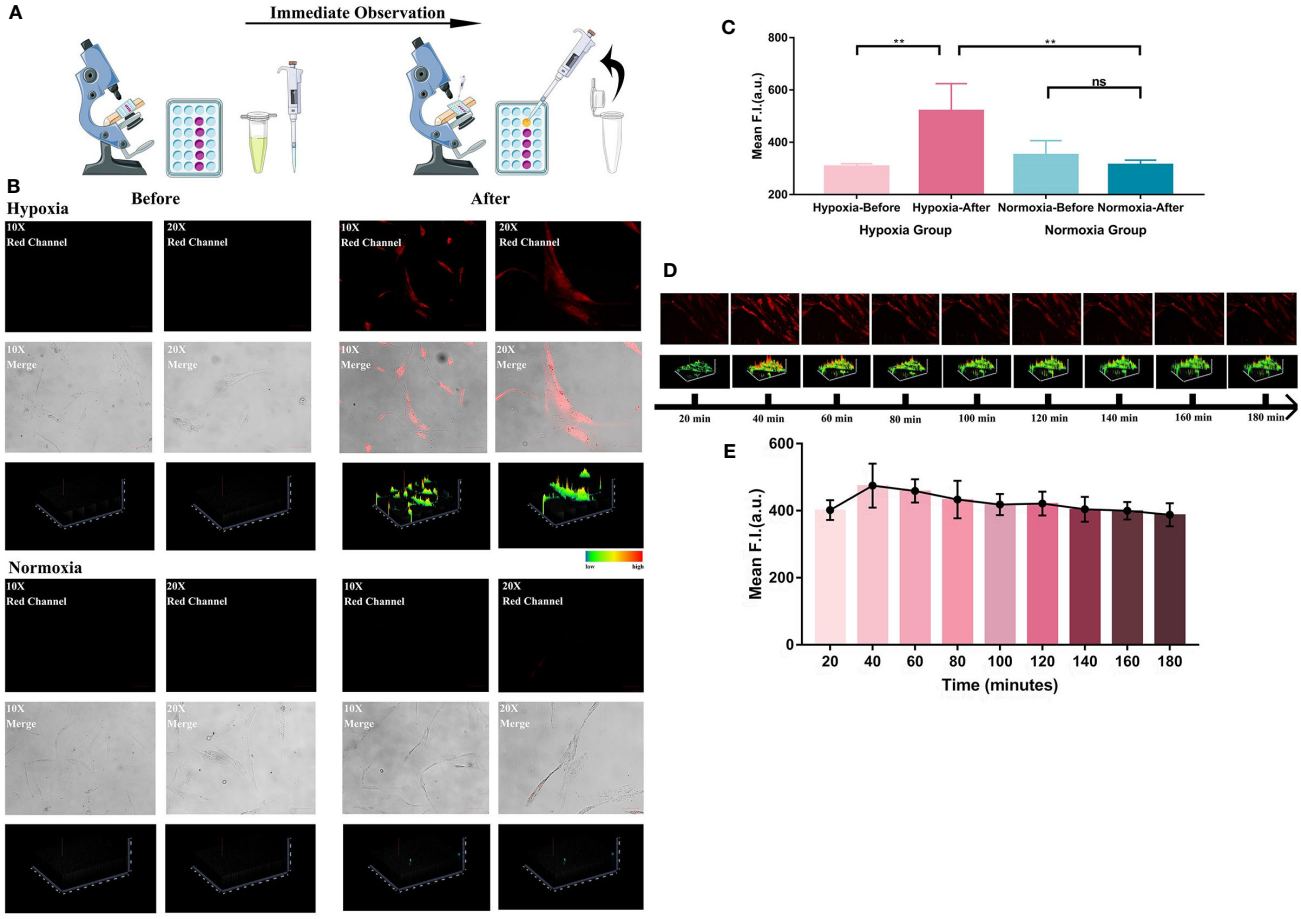
**(A)** Systematic illustration of the process of immediate response test. Both hypoxia group and normoxia group were firstly observed in the microscope before the treatment of HNT-NTR. After adding HNT-NTR, the cells in the same locations were then observed again immediately for comparison. The experiment is to reflect whether the fluorescent probe could show real-time feedback in cells in hypoxic status. **(B)** Fluorescence images and the corresponding 2.5D heatmaps before and after adding HNT-NTR. An obvious change of fluorescence signal after adding HNT-NTR could be observed in hypoxia group. In contrast, the change of fluorescence signal in normoxia group showed no difference. **(C)** Quantitative analysis on the fluorescence intensity of ROI. Significant difference could be observed immediately with the addition of HNT-NTR in HemECs in hypoxic status (P<0.01) **(D)** Time-dependent images of HemECs and the corresponding 2.5D heatmaps for identification of the photostability. Apparent fluorescence signals could still be observed after 3 hours, indicating the strong photostability of HNT-NTR. **(E)** Quantative analysis on the fluorescence intensity of ROI in different time points. The quantative results identified that HNT-NTR could be used for prolonged fluorescence imaging tests with unsignificant loss of fluorescence intensity even after continuous exposure to laser scanning. Scale bar= 50 μm (10X); 25 μm (20X). **P≤0.01. ns, no significance, P>0.05.

Motivated by the excellent results of hypoxia imaging ability in HemECs, we then attempted to identify the rapid detecting ability of the chemosensor (**Figure 3A**). **Figure 3B** described an obvious change of fluorescence signal after adding HNT-NTR into the medium of HemECs in hypoxic status. In contrast, the change of fluorescence signal in normoxia group was relatively low (no significant difference, p=0.19). According to **Figure 3C**, a significant difference could be observed immediately with the addition of HNT-NTR in hypoxic status (p=0.002), indicating the real-time response of the probe when monitoring the hypoxic microenvironment. Besides, the data showed a significant difference between the normoxia and hypoxia group after adding the probe, hinting that HNT-NTR had a superior ability of intervention and could distinguish the hypoxic status rapidly.

The photostability of the probe was also evaluated. After the incubation of HNT-NTR and the removal of unstained probes, the time-dependent imaging of each position was performed to estimate the duration of fluorescence signal. Notably, even after 3 hours, apparent fluorescence signals could still be observed, according to **Figures 3D, E**. The results demonstrated that HNT-NTR could be used for prolonged fluorescence imaging tests with unsignificant loss of fluorescence intensity even after continuous exposure to laser scanning.

### Fluorescence imaging of 3D hemangioma microspheres using HNT-NTR

**Figure 4:**
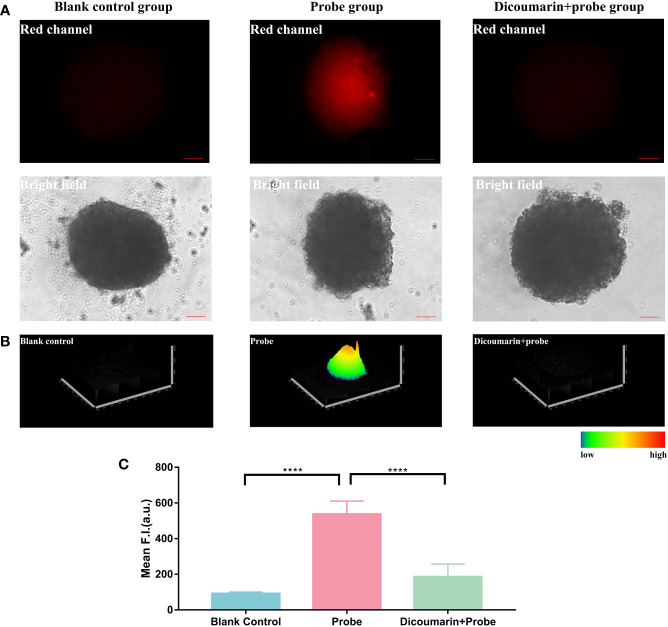
**(A)** Fluorescence imaging of 3D hemagioma microspheres. Blank control group was treated with pure ECM to reduce the autofluorescence of the microspheres; Probe group was treated with only HNT-NTR; Dcioumarin group was prestreated with dicoumarin and then incubated in HNT-NTR to inhibit NTR. **(B)** 2.5D heatmaps of the fluorescence signal. The strongest fluorescence signal could be found in the core of 3D microsphere, in contrast with the weaker signal in the surrounding area. **(C)** Quantitative analysis on the fluorescence intensity of each group. Significant difference could be found between the probe group and the dicoumarin blockage group, indicating the successful development of hemangioma 3D model and demonstrating that HNT-NTR could monitor hypoxia condition in 3D microsphere with the response to NTR. Scale bar= 100 μm. ****P≤0.0001.

After the auspicious application in 2D HemECs, we further constructed a 3D microsphere model for better evaluation of the probe. 3D culture and micro-spheroids have been regarded as effective tools *in vitro* for the simulation of microenvironments and preclinical drug screening (26). Researchers have applied 3D microspheres in evaluating tumor progression and drug delivery (27). 3D microspheres were also used as an effective model for fluorescence visualization *in vitro (* 28, 29). We seeded HemECs in 96-well round bottom ultralow attachment spheroid microplate at a density of 1×10^5^ cells/mL. After being precultured at hypoxia atmosphere for 48 hours, microspheres were then treated with dicoumarin and probes or only probes respectively. As shown in **Figure 4A**, a strong fluorescence signal was detected in probe group. And when pretreated with dicoumarin, the fluorescence signal was substantially inhibited, which also accorded with the result in 2D monolayer culture.

Besides, according to **Figure 4B**, the 2.5D heatmap of the fluorescence imaging, the strongest fluorescence signal was observed in the core of the 3D microsphere, in contrast with the weaker signal in the surrounding area. In light of the opinion of Zhang’s group, 3D microspheres have a core with lower oxygen concentration and a surface with higher concentration (22). Consequently, the NTR-responsive chemosensor could detect the core of the microsphere in higher fluorescence signal when compared to the surface area. We further analyzed the fluorescence intensity of the 3 groups and identified the significant difference between the probe group and the dicoumarin blockage group (**Figure 4C**). The results above indicated the successful development of the 3D hemangioma microsphere model and demonstrated that HNT-NTR could visualize hypoxic status in 3D microspheres with an NTR-response strategy.

### Biosafety and biocompatibility of HNT-NTR

**Figure 5:**
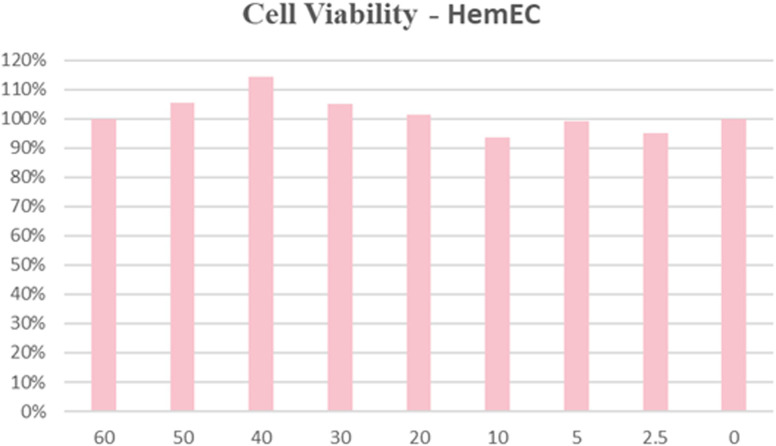
Biocompatibility and biosafety test of HNT-NTR. The cell viability was over 90% in HemECs within 60 μM, indicating the excellent biocompatibility of HNT-NTR.

In previous studies, we have evaluated the biosafety of HNT-NTR in tumor cell lines. In this study, we measured the cytotoxicity of this probe to HemECs. To minimize the interference of solvent (DMSO), we selected 4 mM mother liquid for experiment. We pretreated HemECs with probes in different concentrations (0, 2.5, 5, 10, 20, 30 40, 50, 60 μM) for 24 hours and used CCK-8 assay to measure the cell viability. The result in **Figure 5** showed that even pretreated with 60 uM HNT-NTR, the cell viability was over 90% in HemECs. The result indicated the excellent biosafety and biocompatibility of HNT-NTR.

### *In vivo* identification of the response strategy of HNT-NTR

Encouraged by the favorable results in 2D monolayer culture and 3D microspheres, we then applied HNT-NTR for *in vivo* imaging using a zebrafish model. We tried to further identify the response strategy of HNT-NTR. Zebrafish was a prominent vertebrate model which was widely applied in the detection of drug delivery and fluorescent probe (30, 31), due to its features such as body transparency, simple and rapid construction, and small size (31). We incubated HNT-NTR with zebrafish in 3 different groups (dicoumarin group, probe group and blank control group) to identify the visualization ability *in vivo*.

**Figure 6:**
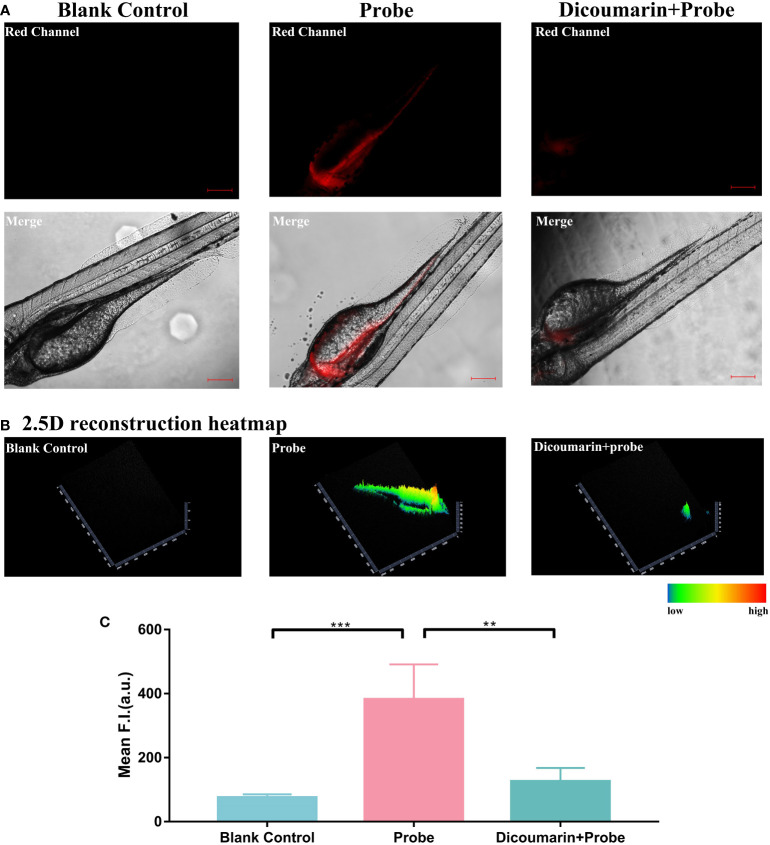
**(A)** Fluorescence imaging of zebrafish. Zebrafish in blank control group were cultured in E medium; in probe group,they were treated with only HNT-NTR; and in dicoumarin group, they were pretreated with dicoumarin to scavenge NTR and then treated with HNT-NTR. With the blockage of dicoumarin, the fluorescence signal was evidentially inhibited compared to the probe group **(B)** The 2.5D heatmaps corresponded to each group. The strongest signal could be found in probe group and weaker signals could be observed in both dicoumarin group and blank control group. **(C)** Quantitative analysis on the fluorescence intensity of each group. Significant difference was identified between the dicoumarin blockage group and the probe group, demonstrating that HNT-NTR has the potential to monitor NTR level in vivo with high efficiency. Scale bar= 100 μm. **P≤0.01. ***P≤0.001.

The results demonstrated that with the blockage of dicoumarin, the fluorescence signal was evidentially inhibited when compared to the probe group, according to **Figure 6A**. We also transformed the fluorescence signal in a 2.5D heatmap and identified the strongest signal in probe group and the weak signal both in dicoumarin group and blank control group, according to **Figure 6B**. A quantitative analysis of ROI was also carried out in **Figure 6C**. According to the results, the probe group showed the highest fluorescence signal (382.4 ± 48.74) compared to both the dicoumarin group (75.22 ± 4.588, *P*=0.0002) and the blank control group (125.6 ± 18.94, *P*= 0.0012) with significant differences. Therefore, HNT-NTR has the potential to monitor NTR levels *in vivo* with high efficiency. The results made a further step to identify the response strategy and the imaging ability of this probe. However, it is difficult for us to translate HNT-NTR into practice for now because of the strict restrictions in ethics. Besides, most of the achievements reported on fluorescent probes for now concentrate on the difference of fluorescence intensity after diverse treatments (28, 32, 33). Some parameters clinicians concentrating on such as false positive rates, false negative rates, and accuracy could not be calculated at this stage. Further experiments on this promising chemosensor would be conducted and the clinical parameters would be measured in the future.

### Modification of the hypoxic status during PRN therapy and the fluorescence imaging using HNT-NTR

Motivated by the excellent fluorescence imaging ability of HNT-NTR, we then attempted to expand the potential application of this probe on changes in the hypoxic status in HemECs during drug therapy. Because one of the first-line treatment drugs of IH, PRN, can suppress IH proliferation, migration and tube formation through HIF-1α dependent pathway (17), and the expression of HIF-1α is the essential biomarker in cells in hypoxia status (20). We supposed that HNT-NTR could visualize the curative efficiency of PRN through the hypoxia-activatable capability. We firstly incubated HemECs with PRN in different concentrations (50 μM, 10 μM, and 0 μM) for 1 hour. After being washed with PBS 3 times, 40 μM HNT-NTR was added to evaluate the fluorescence signal. DAPI was used to locate the cells during fluorescence imaging.

**Figure 7:**
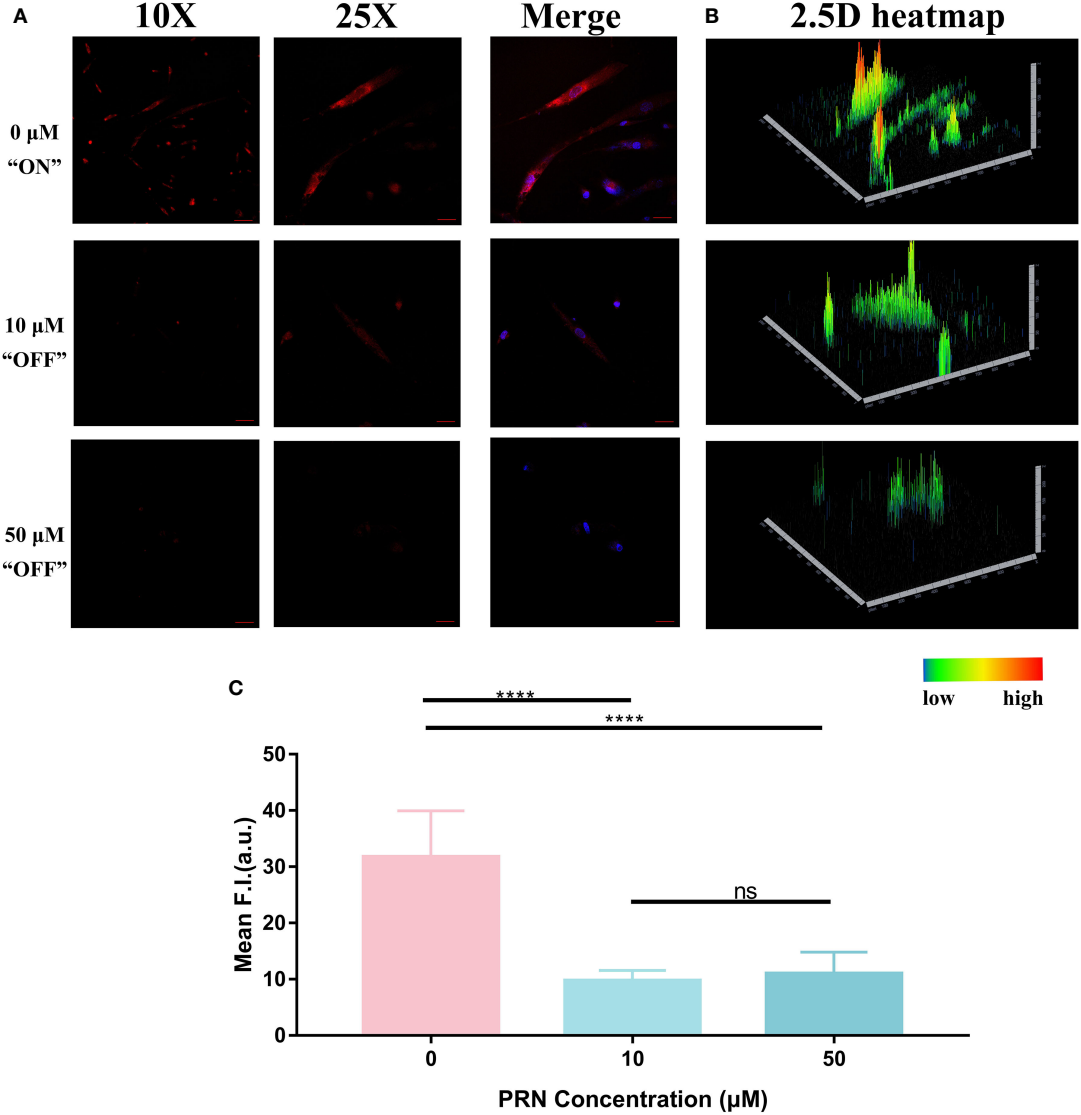
**(A)** Fluorescence imaging of HemECs pretreated with different concentrations of PRN (50 μM, 10 μM, and 0 μM group). HemECs were treated with PRN in different concentrations (50 μM, 10 μM, and 0 μM) for 1 hour. After washed by PBS for 3 times, 40 μM HNT-NTR was added to evaluate the fluorescence signal. DAPI was used to locate the cells. When compared to 0 μM, the other 2 groups exhibited weaker fluorescence signal, indicating that PRN could modify the hypoxic status of HemECs and inhibit the fluorescence signal of HNT-NTR. **(B)** The 2.5D reconstruction heatmaps corresponded to each group. The 2.5D heatmap of PRN treated group exhibited lower bars and more green/dark area compared to the control group. **(C)** Quantitative analysis on the fluorescence intensity of each group. The 0 μM group exhibited significantly higher fluorescence signal compared with the other PRN treated groups (P≤0.0001). Besides, the fluorescence intensity of 0 μM group was approximately 3 times as high as the other 2 groups, indicating that PRN could modify the hypoxic status of HemECs at low concentrations (≤10 μM) and HNT-NTR could visualize modification of hypoxic status during PRN therapy with high sensitivity. Scale bar= 100 μm in 10X image. Scale bar= 40 μm in 25X image. ****P≤0.0001. ns= no significance, P>0.05.

As shown in **Figure 7A**, the fluorescence intensity of 0 μM group (31.74 ± 2.735 in 0μM group) was nearly 3-times as high as the other 2 groups (9.726 ± 0.6154 in 10μM group and 10.97 ± 1.276 in 50μM group), indicating that PRN could modify the hypoxic status of HemECs and inhibit the fluorescence signal of HNT-NTR. The 2.5D reconstruction heatmaps in **Figure 7B** further demonstrated that fluorescence intensity was much lower in PRN treated group. We next quantified the FI of the ROI area. According to **Figure 7C**, the 0 μM group exhibited a significantly higher fluorescence signal compared with the other 3 PRN treated groups (*P ≤* 0.0001). Besides, the fluorescence intensity of 0 μM group was approximately 3 times as high as the other 3 groups (31.74 ± 2.735 in 0 μM group vs. 9.726 ± 0.6154 in 10 μM group, 10.97 ± 1.276 in 50 μM group).

According to the previous study from Gosain’s group, the inhibition of HIF-1α showed a significant difference when treated with 300 μM PRN for 48 hours and 96 hours with conventional techniques such as PCR (19). Some other studies also reflected the modification of the hypoxic status during PRN therapy by detecting the expression of the essential biomarkers such as HIF-1α in cells. The results above demonstrated that PRN could modify the hypoxic status of HemECs at low concentrations (≤10μM) and HNT-NTR could visualize the treatment of PRN with high sensitivity (within 10 μM and 1 hour). HNT-NTR might become an alternative method on detecting the hypoxic status during PRN therapy in substitution for traditional techniques. However, it was a pity that PRN in different concentrations showed no significant difference (*P*=0.39), which indicated that HNT-NTR could only reflect the treated status of PRN in a “Turn-off” sensing method instead of monitoring the measured concentrations of PRN in living cells.

## Conclusion

Remarkably, we successfully visualized the PRN treatment in HemECs using a novel fluorescent chemosensor, NHT-NTR, by “Turn-off” sensing method in this article. In summary, PRN could modify the hypoxic status of HemECs at low concentrations (≤10 μM), and the novel chemosensor NHT-NTR could visualize such a modification with high sensitivity and selectivity during PRN therapy. We also identified that HNT-NTR could visualize the hypoxic status with high sensitivity and selectivity in 2D monolayer HemECs. The fluorescence imaging in 3D hemangioma microspheres and zebrafish also demonstrated the hypoxia-activable mechanism of HNT-NTR with an NTR-responsive strategy. The immediate imaging test demonstrated fast responsiveness and the long duration of fluorescent signal (≥3 hours) was also identified.

Although HNT-NTR showed sensitive NTR-responsiveness, hypoxia-activable ability and could detect hypoxic status in hemangioma cells, there were still some limitations of this chemosensor requiring further investigation: 1) When monitoring the treatment status of PRN in living HemECs, HNT-NTR could only reflect whether the cells were treated by PRN instead of reflecting the concentrations of PRN with a ratiometric method. 2) HNT-NTR is a fluorescent probe designed for the detection of hypoxia and the evaluation of NTR level originally; that is to say, it’s not a specific fluorescent sensor for PRN responsiveness. The application of HNT-NTR in PRN-treatment detection requires further investigations to identify the underlying mechanism whether the enhanced fluorescence signal was affected by the hypoxic status PRN triggered instead of any other influences HemECs reflected. 3) Hindered by limited experimental equipment and strict restrictions in ethics, it’s still difficult for HNT-NTR to apply to patients with hemangioma at present. Further experimental models such as hemangioma-bearing nude mice or large animal models should be testified before clinical trials for the purpose of identifying the biosafety of HNT-NTR. Altogether, HNT-NTR is a novel chemosensor with the potential of application, yet needs further research. Admittedly, we paved a new way for optical imaging of the hypoxic status in HemECs with a novel fluorescence chemosensor.

## Data availability statement

The raw data supporting the conclusions of this article will be made available by the authors, without undue reservation.

## Ethics statement

The animal study was reviewed and approved by the Committee for Ethics of Animal Experiments of Shandong Provincial Hospital Affiliated to Shandong First Medical University.

## Author contributions

Research design: SH, ZC and XW. Experiments performation: YW and XY. Data analysis: MZ, YC, and XL. Manuscript drafting: YW, XY and MZ. Schemes/ Figureures design: JJ, GW, HZ and ZZ. Manuscript review: SH and BZ. All authors contributed to the article and approved the submitted version.

## Funding

1. Shandong Provincial Natural Science Foundation (ZR2021MH270); 2. Jinan Clinical Medical Science and Technology Innovation Plan (202134035); 3. Open foundation of Shandong Key laboratory of Oral Tissue Regeneration (SDDX202105); 4. Natural Science Foundation of Shandong Province (ZR2020QH157). 

## Conflict of interest

The authors declare that the research was conducted in the absence of any commercial or financial relationships that could be construed as a potential conflict of interest.

## Publisher’s note

All claims expressed in this article are solely those of the authors and do not necessarily represent those of their affiliated organizations, or those of the publisher, the editors and the reviewers. Any product that may be evaluated in this article, or claim that may be made by its manufacturer, is not guaranteed or endorsed by the publisher.
