# Supplementary material for: Real-time optical imaging of the hypoxic status in hemangioma endothelial cells during propranolol therapy
Source: Front Oncol. 2022 Oct 4;12:995745. doi: 10.3389/fonc.2022.995745 (PMC9577016; doi:10.3389/fonc.2022.995745)
Supplement: Supplementary file 1 [file DataSheet_1.docx]

**Supporting Information**

**Real-time optical imaging of the hypoxic status in hemangioma endothelial cells during propranolol therapy**

**Authors:**

Yue Wu^1^, Xiaojuan Yang^1^, Mingrui Zhai^2^, Yi Chen^1^_,_ Xiaoya Lu^1^, Jiandong Ju^1^, Guanduo Wang^1^, Huanqing Zhang^1^, Zhe Zhang^2^, Baocun Zhu^3^, Xuan Wang^1, 4*^, Zhanwei Chen^1, 4*^, Shengyun Huang^1, 4*^

**Affiliations:**

^1^Department of Oral and Maxillofacial Surgery, Shandong Provincial Hospital, Cheeloo College of Medicine, Shandong University, Jinan, Shandong, 250021, China.

^2^ Department of Orthodontics, School and Hospital of Stomatology, Cheeloo College of Medicine, Shandong University, Jinan, Shandong, 250012, China.

^3^ University of Jinan, Jinan, Shandong,250021, China.

^4^Department of Oral and Maxillofacial Surgery, Shandong Provincial Hospital Affiliated to Shandong First Medical University, Jinan, Shandong, 250021, China.

Shengyun Huang, Zhanwei Chen, and Xuan Wang are corresponding authors.

Yue Wu and Xiaojuan Yang are first authors who contributed equally to this paper.

*E-mail Address：* huangsy28@sdu.edu.cn (Shengyun Huang)

**Table of Contents**

1. Reagents and apparatus.
2. The synthesis process of **HNT-NTR**.
3. Biocompatibility and biotoxicity evaluation of **HNT-NTR** with CCK-8 assay.
4. Biological characteristics of HemECs.
5. Statistical analysis.
6. Novelty statement.
7. **Reagents and apparatus**

Unless otherwise stated, chemical reagents were purchased from commercial vendor and were used as received. High resolution mass spectra (HRMS) were carried out using a LC-MS2010A instrument. The absorbance of CCK-8 assay was measured by Biological Enzyme Calibration System (ThermoMultiskan GO). The fluorescent imaging of 2D cell culture, 3D microsphere, and zebrafish were observed by either inverted confocal fluorescence microscopy (Leica TCS SP8 SR) or ZEISS inverted microscope (Zeiss Axio Vert, Germany).

1. **The synthesis process of HNT-NTR.**


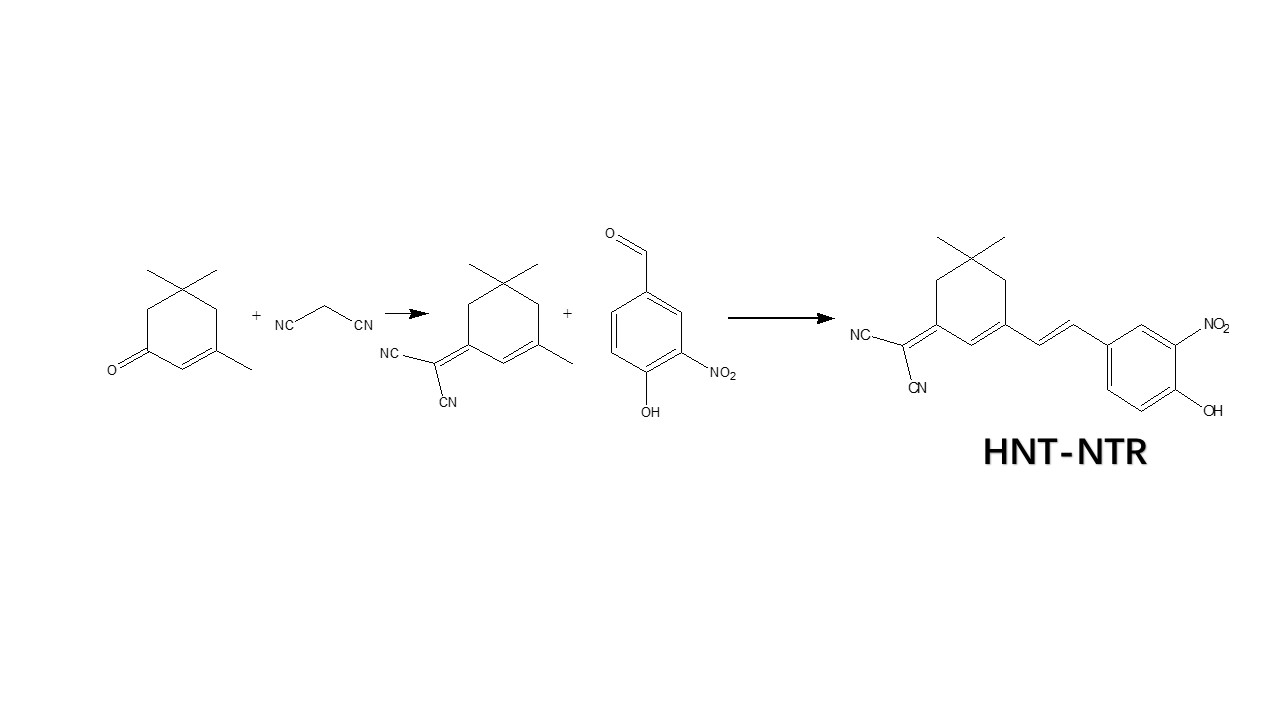


^1^H NMR (400 MHz, CDCl3) d (ppm): 1.10 (s, 6H), 2.47 (s, 2H), 2.63 (s, 2H), 6.94–6.98 (m, 2H), 7.08–7.16 (m, 2H), 7.23 (s, 1H), 8.13 (d, J = 8 Hz, 1H), 10.66 (s, 1H); 13C NMR (100 MHz, CDCl3) d (ppm): 28.0, 32.1, 39.2, 42.9, 81.1, 112.2, 113.0, 118.4, 118.5, 125.8, 126.0, 133.4, 134.1, 144.7, 151.9, 155.4, 168.6. HRMS (ESI): Calcd for C19H16N3O3 [M_H]- 334.1197 Found, 334.1192.

1. **Isolation and culture of hemangioma endothelial cells (HemECs).**

The Isolation and Culture process of primary hemangioma endothelial cells (HemECs) referred to methods mentioned on the previous studies(1, 2). The cells were isolated from resected surgical specimens of infantile hemangiomas. Isolated hemangioma tissue was firstly morcellized and migrate outward in explant culture. Cells were then cultured in endothelial cell medium, supplemented with 10% fetal bovine serum (FBS), 1% penicillin-streptomycin, and 1% EGF.


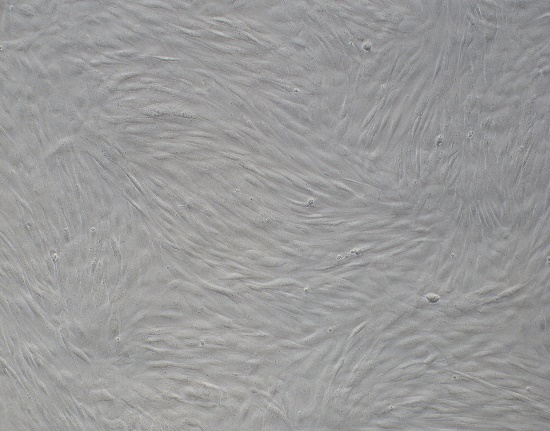

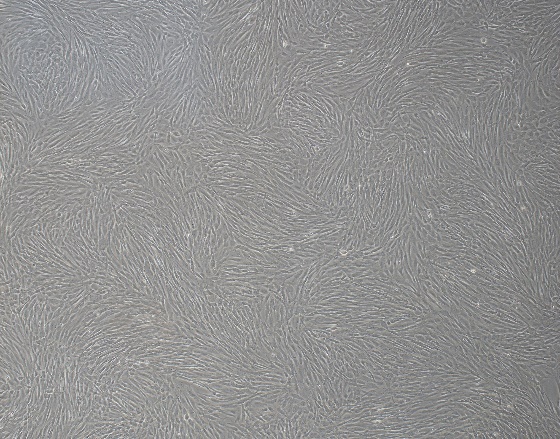


**10X**

**4X**

1. **Biocompatibility and biotoxicity evaluation of HNT-NTR with CCK-8 assay.**

At first, HemECs (3×10^3^) were incubated in 96-well plates for 2 days and then incubated with **HNT-NTR** in different concentrations (0, 2.5, 5, 10, 20, 30 40, 50, 60 μM) for 24 hours at 37 ℃ under 5% CO_2_. After washed with PBS for 3 times, 100 μL of 10% CCK-8 was added. After 1 hour, the absorbance was measured by Biological Enzyme Calibration System (ThermoMultiskan GO). The calculations of living cells were based on the OD values at 450 nm. The formula below was used to calculate the viabilities of cells:

Cell Viability (%) = (Mean OD of various concentrations- Mean OD of blank control)/ (Mean OD of 0 μM group- Mean OD of blank control) ×100%.

1. **Statistical analysis**

All the data of F.I. were analyzed with ImageJ or Zen Blue Lite presented as mean± SD. Mean fluorescent intensity of each ROI was listed and analyzed. Statistical analysis was performed using two-tailed Student’s t-test (unpaired) in GraphPad Prism 7 statistical software. Statistical significance considered as: difference: *P*≤0.05; significant difference: *P*≤0.01.

1. **Novelty statement.**
2. **HNT-NTR can detect the modification of hypoxic status in HemECs during PRN therapy at low concentrations.**

PRN can suppress IH proliferation, migration and tube formation through HIF-1α dependent pathway(3), and the expression of HIF-1α is the essential biomarker in hypoxia condition(4). **HNT-NTR** could visualize the modification of hypoxic status in HemECs during PRN therapy with high sensitivity (within 10 μM and 1 hour). Although the underlying mechanism needs further investigation, we believe that **HNT-NTR** might become an alternative method on PRN therapy detection in substitution for traditional techniques.

| **Experimental technique** | **PRN concentration** | **Treated time** | **Reference** |
| --- | --- | --- | --- |
| Fluorescent probe **HNT-NTR** | 10 μM | 1 h | This investigation |
| Western blotting | ≥50 μM | ≥48 h | (3) |
| Western blotting | ≥100 μM | ≥48 h | (5) |
| ELISA | >50 μM | =24 h | (5) |

1. **We propose a new experimental equipment with NTR-responsive, hypoxia-activable fluorescent chemosensor to detect hypoxic status in hemangioma cells.**

As far as we know, most of the existing experiments elucidating the potential pathogenic linkage between hypoxia and hemangioma at present used conventional methods like real-time RT-PCR, western blotting, and immunohistochemistry to evaluate hypoxia-related molecular markers. Herein, we propose a new experimental method with NTR-responsive fluorescent probe to detect hypoxia condition in hemangioma cells.

| **Experimental methods** | **Detecting Principle** | **Reference** |
| --- | --- | --- |
| Fluorescent probe **HNT-NTR** | Overexpressed NTR in hypoxia condition | This investigation |
| Flow cytometry, Rt-PCR | GLUT-1 | (6) |
| Rt-PCR, Western blotting | HIF-1α | (2) |
| Immunostaining, immunoblotting, ELISA | CCN1 | (7) |

1. **HNT-NTR is the first NTR-responsive fluorescent probe applied in the visualization of hypoxic status in HemECs.**

For all we know, most of the NTR-responsive, hypoxia-activable fluorescent probes were testified in the models of malignant tumors,such as mammary cancer, astrocytoma and hepatocellular carcinoma(8-10).This is because compared to normal tissues, malignant tumors showed an obvious heterogeneity in microenvironment and were tend to a more abnormal metabolism(11, 12). However, IH is a benign microvascular tumor of infancy, for which none of the reported NTR-responsive fluorescent probe was applied in this disease. HNT-NTR is the first NTR-responsive fluorescent probe applied in the visualization of hypoxic status in HemECs.

| **Fluorescent probe** | **Disease model** | **Application** | **Reference** |
| --- | --- | --- | --- |
|  | Infantile Hemangioma | HemECs | This investigation |
|  | Mammary cancer | 4T1 cell line (murine) | (8) |
|  | Astrocytome | U87, U251 cell lines | (9) |
|  | Hepatocellular carcinoma | SMMC-7721 cell line | (10) |

**References**

1. Boye E, Yu Y, Paranya G, Mulliken JB, Olsen BR, Bischoff J. Clonality and altered behavior of endothelial cells from hemangiomas. J Clin Invest. 2001;107(6):745-52.

2. Chen J, Wu D, Dong Z, Chen A, Liu S. The expression and role of glycolysis-associated molecules in infantile hemangioma. Life Sciences. 2020;259:118215.

3. Chen YZ, Bai N, Bi JH, Liu XW, Xu GQ, Zhang LF, et al. Propranolol inhibits the proliferation, migration and tube formation of hemangioma cells through HIF-1α dependent mechanisms. Brazilian Journal of Medical and Biological Research = Revista Brasileira de Pesquisas Medicas E Biologicas. 2017;50(12):e6138.

4. Shu S, Wang Y, Zheng M, Liu Z, Cai J, Tang C, et al. Hypoxia and Hypoxia-Inducible Factors in Kidney Injury and Repair. Cells. 2019;8(3).

5. Chim H, Armijo BS, Miller E, Gliniak C, Serret MA, Gosain AK. Propranolol induces regression of hemangioma cells through HIF-1α-mediated inhibition of VEGF-A. Ann Surg. 2012;256(1):146-56.

6. Herbert A, Ng H, Jessup W, Kockx M, Cartland S, Thomas SR, et al. Hypoxia regulates the production and activity of glucose transporter-1 and indoleamine 2,3-dioxygenase in monocyte-derived endothelial-like cells: possible relevance to infantile haemangioma pathogenesis. Br J Dermatol. 2011;164(2):308-15.

7. Wu P, Xu H, Li N, Huo R, Shen B, Lin X, et al. Hypoxia-Induced Cyr61/CCN1 Production in Infantile Hemangioma. Plast Reconstr Surg. 2021;147(3):412e-23e.

8. Zhang S, Chen H, Wang L, Qin X, Jiang B-P, Ji S-C, et al. A General Approach to Design Dual Ratiometric Fluorescent and Photoacoustic Probes for Quantitatively Visualizing Tumor Hypoxia Levels In Vivo. Angewandte Chemie (International Ed In English). 2022;61(7):e202107076.

9. Klockow JL, Hettie KS, LaGory EL, Moon EJ, Giaccia AJ, Graves EE, et al. An Activatable NIR Fluorescent Rosol for Selectively Imaging Nitroreductase Activity. Sens Actuators B Chem. 2020;306.

10. Zheng A, Sun H, Du Y, Wang Y, Wu M, Liu X, et al. A novel long-wavelength off-on fluorescence probe for nitroreductase analysis and hypoxia imaging. Anal Chim Acta. 2021;1144:76-84.

11. Dagogo-Jack I, Shaw AT. Tumour heterogeneity and resistance to cancer therapies. Nature Reviews Clinical Oncology. 2018;15(2):81-94.

12. Martínez-Reyes I, Chandel NS. Cancer metabolism: looking forward. Nature Reviews Cancer. 2021;21(10):669-80.
